# Voxel-based morphometry and functional connectivity changes are associated with cognitive function in herpes simplex virus encephalitis

**DOI:** 10.3389/fnins.2025.1714446

**Published:** 2026-01-12

**Authors:** Qinglong Feng, Yan Li, Zhipeng Xu, YanDan Xu, Jueyue Yan, XiongYan Lan, Xiaofen Zhu

**Affiliations:** 1 Intensive Care Unit, QuZhou KeCheng People’s Hospital, Quzhou, Zhejiang, China; 2 Department of Critical Medicine, The People’s Hospital of Pingyang, Wenzhou, Zhejiang, China; 3 Department of Critical Care Medicine, The First Affiliated Hospital, Zhejiang University School of Medicine, Hangzhou, Zhejiang, China; 4 Intensive Care Unit, The Second People’s Hospital of Quzhou, Quzhou, Zhejiang, China; 5 Department of Neurology, QuZhou KeCheng People’s Hospital, Quzhou, Zhejiang, China

**Keywords:** cognitive impairment, functional connectivity, herpes simplex encephalitis, interleukin-6, neuroinflammation, voxel-based morphometry

## Abstract

**Purpose:**

Herpes simplex encephalitis (HSE) is a severe neurological condition associated with significant cognitive impairment and structural brain changes. This study aimed to investigate microstructural and functional connectivity (FC) alterations in HSE patients and their association with cognitive function, cerebrospinal fluid (CSF) parameters, and inflammatory markers.

**Methods:**

A single-center cohort study was conducted with 73 HSE patients and 76 cognitively unimpaired controls. Voxel-based morphometry (VBM) and resting-state functional MRI (rs-fMRI) were used to assess VBM grey matter volume (GMV) and FC. Cognitive function was evaluated using the Montreal Cognitive Assessment (MoCA). CSF pressure, protein levels, and proinflammatory cytokines (IL-6, IL-1β, IL-2, IL-4, IL-5, IL-10) were measured. Statistical analyses included group comparisons and multivariable regression adjusted for age, gender, and hypertension.

**Results:**

HSE patients exhibited significant GMV reductions in the right hippocampal gyrus, left precuneus, and left posterior cingulate gyrus (all *p* < 0.001). Enhanced FC was observed between the left hippocampus and medial prefrontal cortex (mPFC), while weakened connectivity was noted between the left precuneus, posterior cingulate gyrus, and mPFC in controls. Cognitive scores (MoCA) were lower in HSE patients (*p* < 0.001) and positively correlated with GMV and FC metrics (*p* < 0.05). Elevated CSF pressure, protein, and proinflammatory cytokines (particularly IL-6) were negatively associated with cerebral metrics (*p* < 0.001). A significant interaction between IL-6 and cerebral metrics further influenced cognitive outcomes (*p* < 0.05).

**Conclusion:**

HSE is associated with distinct microstructural and functional connectivity changes that are correlated with cognitive impairment and neuroinflammation. Our findings suggest a potential interaction between IL-6 levels, cerebral alterations, and cognitive dysfunction, which may inform the exploration of neuroimaging and inflammatory biomarkers in personalized therapeutic strategies. However, these represent observational associations, and further prospective studies are needed to validate these findings and establish causal relationships.

## Introduction

Herpes simplex encephalitis (HSE) represents the most frequent recognized etiology of sporadic necrotizing encephalitis across the globe (Cleaver et al., 2024; Armangue et al., 2023; Granerod et al., 2010). Without therapy, HSE carries an extremely poor prognosis, with mortality approaching 70%. Advances in diagnosis and treatment have reduced mortality to ~15%; however, substantial disability remains common among survivors (Backman et al., 2015). Early, reliable prognostic markers in HSE could fundamentally improve tailored treatment strategies. Despite this, robust data on outcomes such as cognitive impairment in severe HSE are lacking, and much of the literature is constrained by limited sample sizes.

The advancement in neuroimaging technology has allowed structural as well as functional information to be provided on the brains of HSE patients. A voxel-based morphometry (VBM) analysis analyzes voxel-wise differences in the local morphology of the brain between groups. This is a whole-brain morphology analysis that compares voxel-wise differences. According to neuroimaging studies, HSE cerebral microstructural volume differs significantly from that of controls (Gitelman et al., 2001; Frisch et al., 2015; Ono et al., 2009). A powerful and versatile tool for studying noninvasive functional activation of the human brain *in vivo* is functional magnetic resonance imaging (fMRI) using a blood oxygenation level-dependent (BOLD) signal. In resting-state functional MRI (RS-MRI), functional connectivity (FC) is a measure of blood-oxygen-level-dependent signals in different regions of the brain over time; seed correlation analysis is one method for assessing FC.

Although neuroimaging studies have shown cerebral changes in HSE, very little is known about the cerebral functional changes in HSE and their association with clinical measures such as neuropsychological scores. Our study aimed to address this research gap by exploring the microstructural and functional cerebral changes in HSE. We hypothesized that: (1) HSE patients would exhibit significant reductions in VBM grey matter volume and alterations in functional connectivity in limbic and default mode network regions compared to cognitively unimpaired controls; and (2) these cerebral metrics would be associated with cognitive impairment, CSF parameters, and inflammatory markers, with a potential moderating role of proinflammatory cytokines like IL-6. Our aims are: Investigate the differences in VBM grey matter volume and FC between HSE and cognitively unimpaired controls Association between VBM grey matter and FC metrics with cognitive measures and inflammatory markers in HSE.

## Methods

This single-center cohort was conducted at the First Affiliated Hospital of Zhejiang University School of Medicine (China) from January 2021 to August 2025. We screened patients with a clinical diagnosis of encephalitis [International Classification of Diseases, 9th Revision (ICD-9) code 054.3] and confirmed herpes simplex virus (HSV) in cerebrospinal fluid by polymerase chain reaction. Neuroimaging was performed in all cases. The protocol was approved by the institutional ethics committee of the First Affiliated Hospital of Zhejiang University School of Medicine and adhered to the Declaration of Helsinki. Reporting followed the Strengthening the Reporting of Observational Studies in Epidemiology (STROBE) guidelines.

### HSE patients

Patients were included when all of the following were present: ICU admission with length of stay ≥24 h; possible acute encephalitis per international guidelines (Venkatesan et al., 2013) and a CSF PCR positive for HSV DNA obtained during the hospital course. Possible acute encephalitis was operationalized as an acute mental or behavioral status change lasting ≥24 h plus at least two criteria: (1) fever within 72 h before/after presentation; (2) generalized or focal seizures; (3) new-onset focal neurological deficits; (4) CSF white blood cell count ≥5 cells/μL; (5) neuroimaging or EEG abnormalities indicative of encephalitis. Exclusion criteria comprised missing neuroimaging or imaging performed >14 days after ICU admission, antecedent neurological conditions that could hinder MRI analysis (e.g., brain tumor, severe traumatic brain injury), inadequate MRI quality, or incomplete clinical data.

### Clinical information

Patient histories together with clinical, laboratory, and neuroimaging data were extracted from the medical records. A neuropsychological evaluation was performed at ICU admission. Global cognition was measured with the Montreal Cognitive Assessment (MoCA), a brief screening instrument with a total score of 30, where higher scores denote better cognitive performance (Smith et al., 2007; Dong et al., 2013).

All patients underwent lumbar puncture, and cerebrospinal fluid levels were assessed and measured. Cytokines (IL-10, IL-12, IL-5, IL-1β, IL-2, IL-4, and IL-6) were also assessed and recorded.

Cognitively unimpaired participants (CU) were recruited through advertisements; these individuals were residents who lived around the hospital or attended our hospital for annual health examinations. Participants who denied a history of neurologic disorders, did not show any abnormality on MRI, and were cognitively normal were included in our study. Clinical information was recorded for all controls. Exclusion criteria were in line with the aforementioned criteria.

### Neuroimaging examination

MRI examinations were scheduled during the initial week following ICU entry, and imaging for each participant was acquired on a Siemens Trio Tim 3 T platform with a 32-channel head coil.

High-resolution structural T1-weighted images for voxel-based morphometry (VBM) were acquired using a 3D magnetization-prepared rapid gradient-echo (MPRAGE) sequence with the following parameters: repetition time (TR) = 2,300 ms, echo time (TE) = 2.98 ms, flip angle = 9°, inversion time (TI) = 900 ms, field of view (FOV) = 256 × 256 mm, matrix size = 256 × 256, voxel size = 1 × 1 × 1 mm, number of slices = 176, acquisition time ≈ 5 min.

Resting-state functional MRI (rs-fMRI) data were acquired using a gradient-echo echo-planar imaging (EPI) sequence with the following parameters: TR = 2,000 ms, TE = 30 ms, flip angle = 90°, FOV = 200 × 200 mm, matrix size = 64 × 64, voxel size = 3.125 × 3.125 × 3.5 mm (with 0.6 mm gap), number of slices = 33, number of volumes = 240, acquisition time ≈ 8 min.

### Data processing

For structural MR images, FSL’s standard VBM processing pipeline was implemented. An optimized VBM approach was implemented with all processing steps carried out employing open-source FSL version 4.1.5 (Good et al., 2001). Structural MRI data underwent optimized VBM using FSL v4.1.5. Brain extraction (BET) and tissue segmentation (FAST) were applied to each single-timepoint grey matter image. Images were non-linearly registered to MNI152 space via FNIRT, averaged to create a study-specific template, re-registered, modulated, and smoothed (*σ* = 3 mm). Only one scan was acquired per participant; no session-to-session alignment was required or performed.

All fMRI preprocessing was conducted using Statistical Parametric Mapping version 12 (SPM12; Wellcome Centre for Human Neuroimaging, London) and the Computational Anatomy Toolbox (CAT12) within MATLAB R2020b. The pipeline involved the following sequential steps: the first 10 volumes were discarded to allow for magnetic field stabilization; slice timing correction was applied to account for inter-slice acquisition differences; head motion correction was performed via realignment to the mean image using a six-parameter rigid-body transformation. Structural T1-weighted images were then co-registered to the functional mean image for optimal alignment. Spatial normalization to the Montreal Neurological Institute (MNI) template space was achieved using the unified segmentation approach in CAT12, with data resampled to 2 × 2 × 2 mm (Granerod et al., 2010) voxels. Spatial smoothing was applied with a 6-mm full-width at half-maximum Gaussian kernel. Rigorous quality control included motion scrubbing with a framewise displacement threshold of 0.2 mm, and participants exhibiting excessive head motion (>2 mm translation or >2° rotation) were excluded. The preprocessed data were detrended to remove linear and quadratic drifts, temporally band-pass filtered (0.01–0.1 Hz), and subjected to nuisance regression of 24 motion parameters, white matter and cerebrospinal fluid signals, and their temporal derivatives. Seed-based functional connectivity analysis was then performed by computing Pearson’s correlation coefficients between the mean time series of spherical regions of interest (6-mm radius) and all other brain voxels, which were subsequently converted to *z*-values using Fisher’s transformation for normality.

#### FSL voxel-based morphometry (FSL-VBM)

We performed non-parametric statistical analysis using FSL Randomize with 5,000 permutations and threshold-free cluster enhancement (TFCE) option (*p* < 0.05).

#### Seed-based resting-state functional connectivity analysis

**Figure 1:**
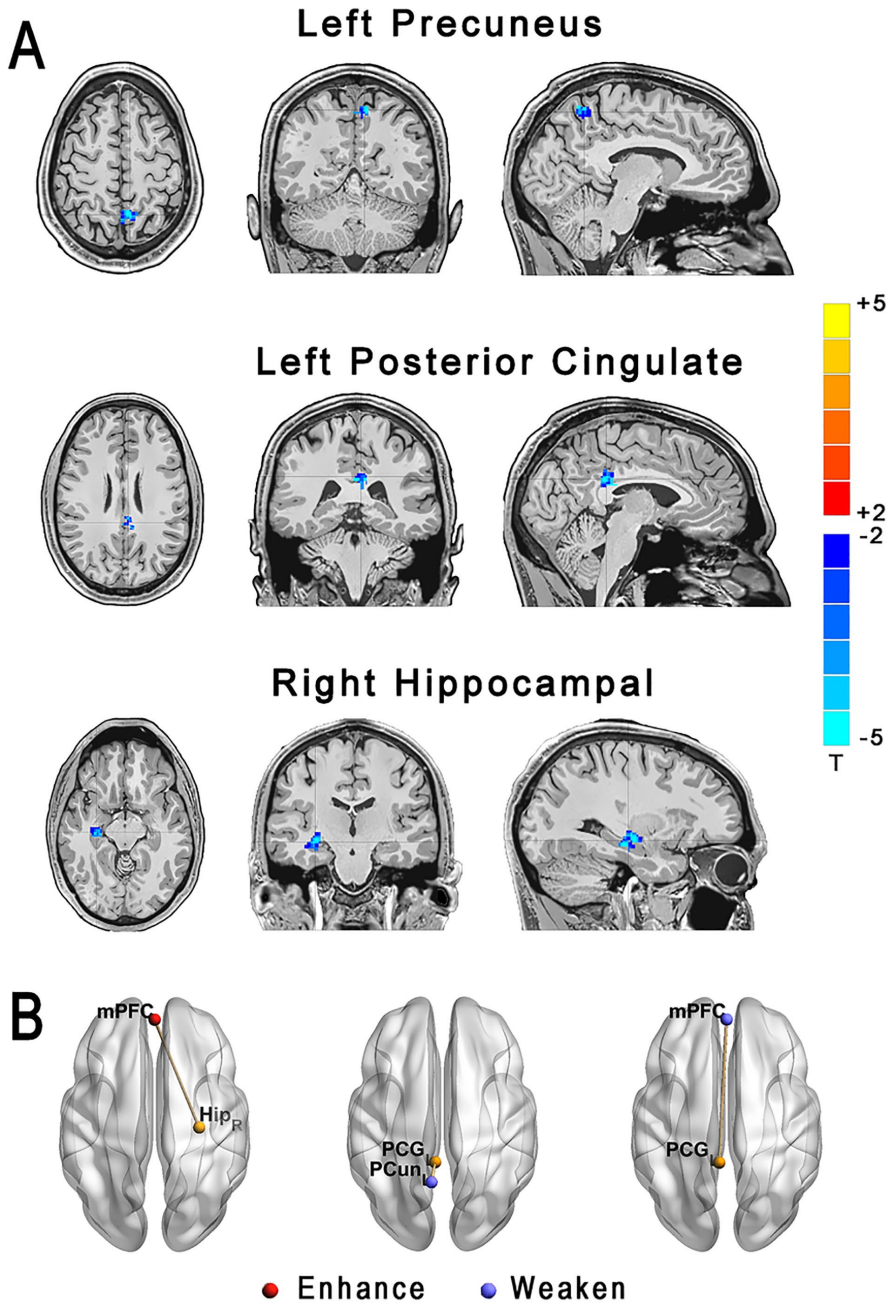
**(A)** VBM maps showing significant GMV reductions in HSE vs. controls: left precuneus (top), left posterior cingulate gyrus (middle), and right hippocampal gyrus (bottom), in axial, coronal, and sagittal views. Color bar: *T*-values (blue for reductions). **(B)** FC alterations: enhanced in HSE between mPFC (red) and left hippocampus (Hip_L, yellow); weakened (vs. controls) between mPFC, left precuneus (PCun_L, red), and left posterior cingulate gyrus (PCG_L, yellow).

A two-tailed *t*-test was used for comparisons of the FC between the groups. Seed regions were data-driven, defined based on the significant VBM findings to focus on areas most affected in HSE: specifically, three seeds were used—the right hippocampal gyrus (MNI coordinates: peak voxel from VBM analysis), left precuneus, and left posterior cingulate gyrus—with ROIs drawn as 6-mm radius spheres centered on peak voxels from the group VBM differences (as shown in Figure 1; Supplementary Figure 2). This approach is exploratory and not *a priori*. These seeds were chosen to investigate connectivity alterations in limbic and default mode network regions implicated in HSE pathology, with anatomical definitions based on the automated anatomical labeling (AAL) atlas (Tzourio-Mazoyer et al., 2002). Correction for multiple comparisons was accomplished with 3D-ClustSim using Analysis of Functional NeuroImages (AFNI). 3D-ClustSim parameters included a voxel-wise *p*-threshold of 0.001, simulation of 10,000 iterations for noise estimation (assuming Gaussian spatial autocorrelation with a smoothness of ~8 mm FWHM based on our data), and a family-wise error (FWE) corrected cluster-level threshold of *p* < 0.05, resulting in a minimum cluster size of 20 contiguous voxels (adjusted for our 3 mm isotropic voxel size and whole-brain mask). *p* < 0.05 was considered statistically significant. All results were viewed on the MNI T1 template, and the *p* or T-value scale is shown on the right of the image.

### Statistical analyses

To examine the differences between controls and HSE, the chi-square test was used for categorical variables, ANOVA for continuous and normally distributed variables, and the Kruskal–Wallis test for skewed data. To examine the association between cerebral metrics and clinical variables in HSE, multivariable linear regression analyses were constructed. All regression analyses were adjusted for age, gender, and hypertension and subsequently for education (for cognitive measures). We report standardized beta coefficients (*β*), 95% confidence intervals (CIs), effect sizes (partial *η*^2^ for interactions), and *p*-values for key associations.

To examine the association between cerebral metrics and clinical variables in HSE, multivariable linear regression analyses were constructed. All regression analyses were adjusted for age, gender, and hypertension and subsequently for education (for cognitive measures). We report standardized beta coefficients (*β*), 95% confidence intervals (CI), effect sizes (partial *η*^2^ for interactions), and *p*-values for key associations. All correlations are pairwise (2-way) Pearson correlations between two variables. We applied false discovery rate (FDR) correction using the Benjamini-Hochberg procedure. No 3-way or partial correlations are displayed. Env-cor is a composite covariate included as a single variable in all pairwise analyses.

We further tested the moderation (interaction) effect between cerebral metrics and laboratory results on cognitive measures by including the cross-product term of “individual cerebral metric × individual laboratory metric” (e.g., IL-6 × GMV) with the main effect terms of each variable in the models. These models were adjusted for age, gender, hypertension, and education. Mediation was not tested, as our hypotheses focused on moderation to assess how inflammatory markers (e.g., IL-6) influence the strength of the relationship between cerebral changes and cognition. For all models, *p*-value < 0.05 was considered statistically significant.

## Results

Supplementary Figure 1 shows the flow chart of our study participants. As summarized in Table 1, 131 participants were enrolled (76 controls and 73 with HSE). The two groups were comparable in baseline demographics: mean age (controls 31.03 ± 6.50 vs. HSE 31.96 ± 7.24 years; *p* = 0.450), proportion of males (75.86% vs. 80.82%; *p* = 0.636), prevalence of hypertension (34.48% vs. 42.47%; *p* = 0.453), and years of education [median (IQR) 12 (9, 12) vs. 12 (9, 12); *p* = 0.348]. In contrast, cognitive performance differed markedly in Table 1, with the HSE group showing a lower MoCA score than controls [median (IQR) 22 (20, 24) vs. 28 (28, 30); *p* < 0.001]. As summarized in Table 1, the two groups were comparable in baseline demographics: mean age (controls 31.03 ± 6.50 vs. HSE 31.96 ± 7.24 years; *p* = 0.450; normal distribution confirmed, Shapiro–Wilk *p* = 0.12 for controls, *p* = 0.18 for HSE), proportion of males (75.86% vs. 80.82%; *p* = 0.636), prevalence of hypertension (34.48% vs. 42.47%; *p* = 0.453), and years of education [median (IQR) 12 (9, 12) vs. 12 (9, 12); *p* = 0.348; skewed, Shapiro–Wilk *p* < 0.05]. Cognitive performance differed markedly, with HSE showing lower MoCA scores [median (IQR) 22 (20, 24) vs. 28 (28, 30); *p* < 0.001; skewed]. HSE patients presented with median temperature 39.0 °C (IQR 39.0–40.0; skewed) and abnormal CSF indices: opening pressure 268.85 ± 47.92 (normal), protein 559.18 ± 96.30 (normal), and cytokines (e.g., IL-6349.14 ± 164.34; normal). The normal range for CSF opening pressure in adults is typically 70–180 mm H_2_O in the lateral decubitus position, while in our HSE cohort, the mean value was 268.85 ± 47.92 mm H_2_O, indicating elevated pressure consistent with neuroinflammation; for CSF protein, the normal range in adults is 15–45 mg/dL with slight increases up to 70 mg/dL in older adults, whereas our HSE patients showed a markedly elevated mean of 559.18 ± 96.30 mg/dL, reflecting the inflammatory pathology; and for CSF proinflammatory cytokines such as IL-6, IL-1β, IL-2, IL-4, IL-5, and IL-10, these are typically undetectable or present at very low levels in healthy adults, with normal IL-6 often less than 5–10 pg/mL, IL-1β less than 2 pg/mL, and similar low thresholds for others like IL-2 less than 5 pg/mL, as they are not routinely elevated without inflammation, in contrast to our HSE group which exhibited significantly higher levels, for example, IL-6 mean 349.14 ± 164.34 pg/mL, underscoring the role of cytokine-mediated neuroinflammation.

**Table 1:** Demographics and clinical characteristics of study participants.

Characteristics	Controls (*n*=76)	HSE (*n*=73)	*p*-value
Age, years	31.03 (6.50)	31.96 (7.24)	0.450
Males, *n* (%)	44 (57.89%)	59 (80.82%)	0.636
Hypertension, *n* (%)	20 (26.31%)	31 (42.47%)	0.453
Education, years	12 (9, 12)	12 (9, 12)	0.348
MoCA	28 (28, 30)	22 (20, 24)	<0.001
Temperature		39 (39, 40)	
Fever (*T* ≥ 38.3°C)		64 (87.67%)	
CSF, pressure		268.85 (47.92)	
CSF, protein		559.18 (96.30)	
IL-6		349.14 (164.34)	
IL-1β		1.86 (1.19)	
IL-2		3.37 (1.52)	
IL-4		2.07 (2.07)	
IL-5		3.31 (1.04)	
IL-10		6.30 (2.04)	
IL-12		1.62 (0.40)	

Data are presented as mean (SD) for normally distributed continuous variables, median (IQR) for skewed continuous variables, or *n* (%) for categorical variables. CSF opening pressure in mm H₂O; CSF protein in mg/dL; interleukins (IL) in pg/mL. MoCA, Montreal Cognitive Assessment; CSF, cerebrospinal fluid; IL, interleukin.

### VBM grey matter volume between HSE and controls

Analyses of VBM GMV showed significantly reduced GMV in the right hippocampal gyrus, left precuneus, and left posterior cingulate gyrus (all *p* < 0.001; Figure 1; Supplementary Figure 2) in HSE compared to controls. MNI coordinates was shown in Supplementary Table 1.

### Comparison of functional connectivity between HSE and controls

We compared the resting-state functional connectivity using various brain regions as seed points. Our results indicated that, in HSE, the connectivity between the left hippocampus (Hip_L) and the medial prefrontal cortex (mPFC) was significantly enhanced. Conversely, in the control group, the connectivity between the left precuneus (PCun_L) and the left posterior cingulate gyrus (PCG_L), as well as between PCG_L and mPFC, was notably weakened, as shown in Figure 1. Supplementary Table 2 shows HSE showed higher FD (*p* = 0.021); FC results robust to motion correction.

**Figure 2:**
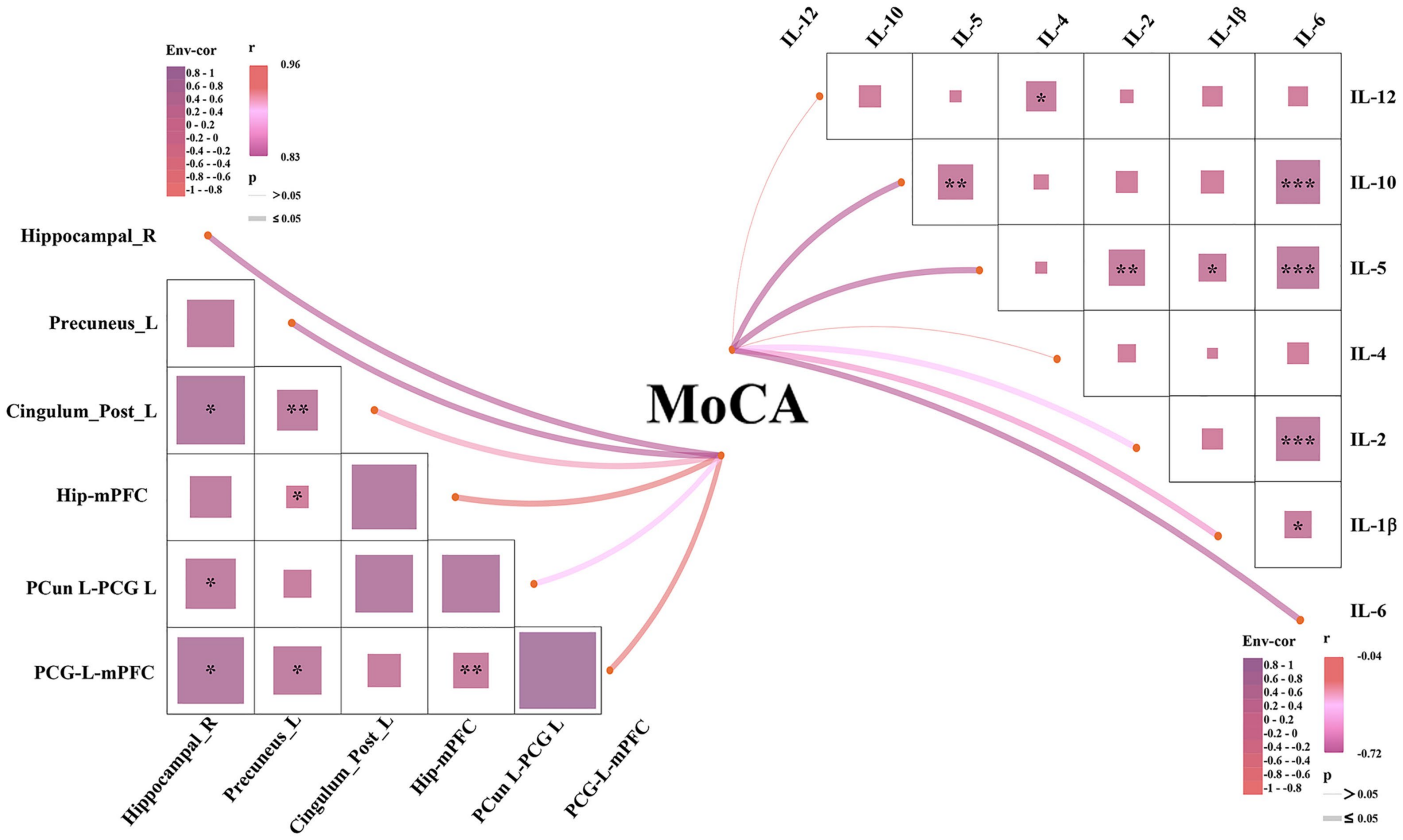
Correlation network between cerebral metrics (GMV and FC), inflammatory cytokines, and cognitive function (MoCA) in HSE patients. Upper panel: Correlation matrix with Pearson *r* values between Env-cor (environmental covariate correlation factor: composite of age, gender, hypertension, fever), brain regions, FC pairs, cytokines (IL-1β, IL-2, IL-4, IL-5, IL-6, IL-10, IL-12), and MoCA. Lower panel: Network visualization with edge color/thickness indicating correlation strength. ^*^p ≤ 0.05, ^**^p < 0.01, and ^***^p < 0.001. The thick lines indicate that the *p* ≤ 0.05, and different colors of the lines represent different correlation coefficients (*r* 0.83–0.96).

Figure 2 shows the relationships between structural brain regions, FC, inflammatory cytokines, and cognitive function in patients with HSE. The figure presents a correlation matrix depicting the associations between VBM grey matter volume, FC between various brain regions, proinflammatory cytokines, and cognitive performance (MoCA). The results showed that MoCA score was significantly correlated with brain volume changes, FC changes and CSF inflammatory factors (p_FDR < 0.05). Information about effect size, 95% confidence interval or regression coefficient are presented in the Supplementary Figure 4.

**Figure 3:**
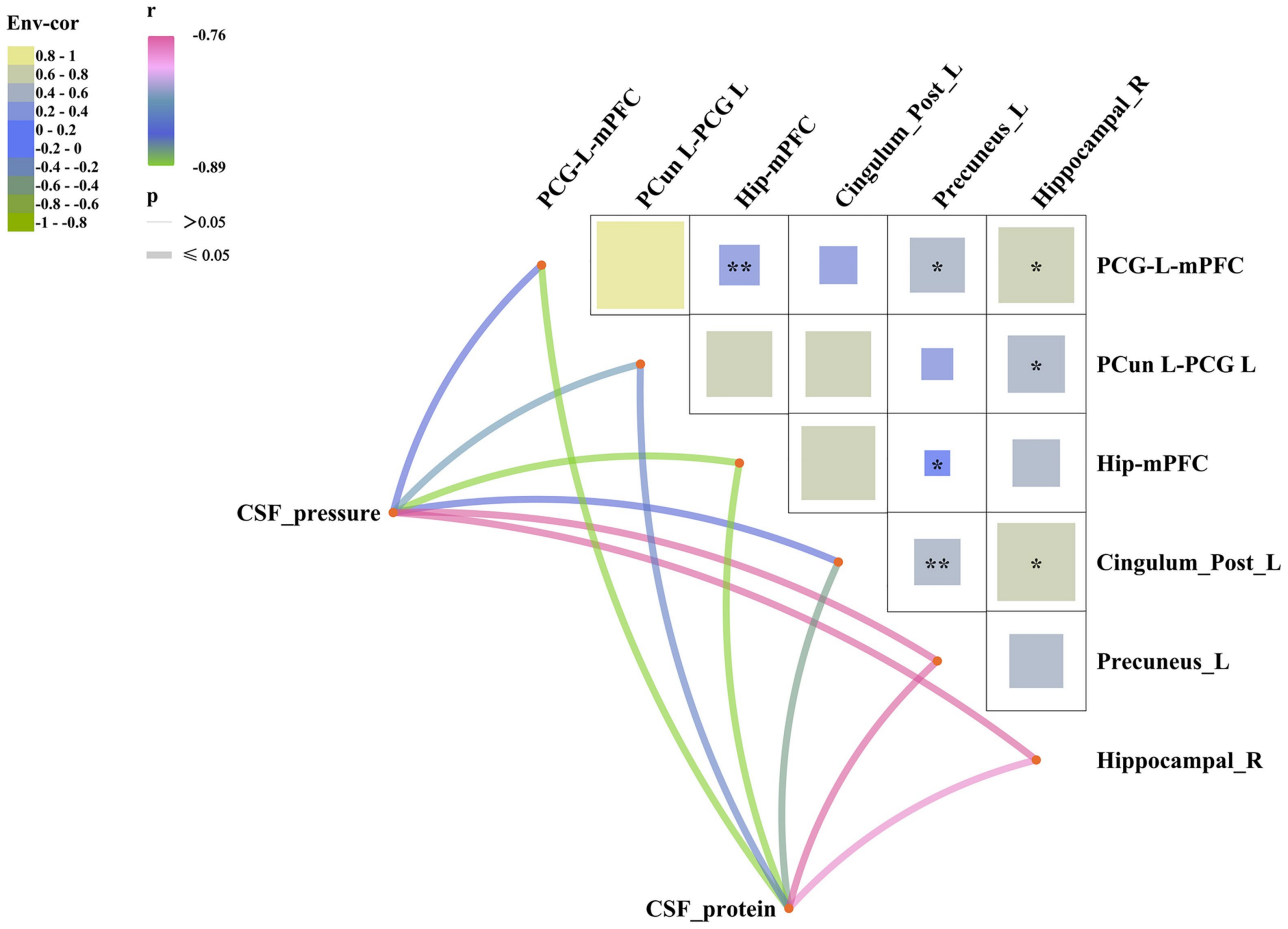
Correlation network between CSF parameters (pressure, protein) and cerebral metrics in HSE patients. Upper panel: Correlation matrix with Pearson *r* values between Env-cor (environmental covariate correlation factor: composite of age, gender, hypertension, fever), CSF measures, GMV (right hippocampus, left precuneus, left posterior cingulate), and FC pairs. Lower panel: Network graph with edge color/thickness indicating correlation strength. Color scale: *r* from −1 (blue) to +1 (red); ^*^p ≤ 0.05, ^**^p < 0.01, and ^***^p < 0.001. The thick lines indicate that the *p* ≤ 0.05, and different colors of the lines represent different correlation coefficients (*r* = −0.89 to −0.76).

Figure 3 shows the relationships between cerebrospinal fluid (CSF) parameters, including CSF pressure and CSF protein levels, with structural brain regions and functional connectivity in patients with herpes simplex encephalitis (HSE). The correlation matrix visualizes how these CSF metrics are associated with brain regions and FC, with *r* values and FDR-corrected *p*-values presented for each pair of variables. We can find that there is a significant correlation between CSF parameters and MRI results (all p_FDR < 0.05). Information about effect size, 95% confidence interval or regression coefficient are presented in the Supplementary Figure 5.

**Figure 4:**
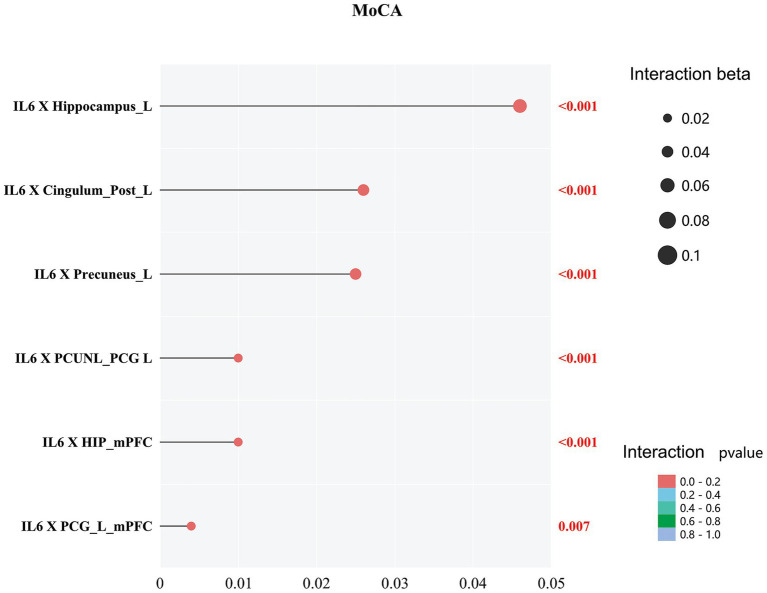
Interaction effects of IL-6 with GMV/FC on MoCA in HSE. Points show standardized *β* coefficients (*x*-axis: 0–0.05) for IL-6 × cerebral metric terms; color indicates *p*-value (red: *p* < 0.001; gradient to *p* = 0.007). All *p* < 0.05.

Importantly, we showed a significant interaction between IL-6 and cerebral metrics (GMV and FC) on cognitive measures (MoCA) in HSE (all *p* < 0.05; Figure 4).

## Discussion

In this cross-sectional study of 73 HSE patients, we observed significant cerebral VBM grey matter volumetric and functional connectivity changes when compared to controls. We also showed that cerebral changes in HSE were associated with their cognitive measures. Importantly, we showed that VBM grey matter and functional connectivity changes and increased IL-6 levels jointly influenced cognitive function in HSE.

Previous reports (Gitelman et al., 2001; Frisch et al., 2015) have shown reduced VBM grey matter volume in HSE compared to controls. Similarly, we showed that voxel-based morphometry analysis showed reduced VBM grey matter volume in the right hippocampal gyrus, left precuneus, and left posterior cingulate gyrus compared to controls, which is in line with previous studies.

fMRI is a neuroimaging technique that indexes neural activity via changes in deoxyhemoglobin, i.e., the blood oxygen level-dependent (BOLD) signal. Two mainstream approaches are used: (1) activation fMRI, which evaluates task-evoked modulation of the deoxyhemoglobin signal; and (2) resting-state fMRI, which quantifies synchrony of low-frequency BOLD fluctuations across regions to infer functional connectivity (FC). In HSE, we observed reduced connectivity in the left posterior cingulate cortex and hippocampus, alongside increased FC in the medial prefrontal cortex relative to controls. To our knowledge, these HSE-related connectivity alterations are reported here for the first time.

The difference in the cerebral regions seen in our HSE and controls is associated with the limbic and paralimbic systems of the brain, shares close anatomic connectivity, and may possess shared immunological markers, making them a common target of HSV (Barnett et al., 1994; Herdman et al., 2015; Kapur et al., 1994). Moreover, previous histopathological studies have shown injury and the presence of viral antigens in these regions (Cleaver et al., 2024; Aldea et al., 2003). Reduced VBM GMV and decreased functional connectivity suggest neurodegeneration in these areas. In response to injury, increased connectivity may indicate efforts at compensatory or noncompensatory network reorganization.

CSF pressure and cerebral microstructural changes are directly related. In this study, we found a negative association between increased CSF pressure and proteins related to VBM and functional connectivity. As the brain is a fixed space, an increase in CSF will compress brain tissue, potentially resulting in cerebral changes.

We also showed that IL-1β, IL-2, IL-4, IL-5, IL-6, and IL-10 were negatively associated with VBM GMV and functional connectivity changes in HSE. In line with our results, previous studies (Jefferson et al., 2007) associated interleukin with decreased total brain volume and cortical atrophy in healthy controls. These negative associations may explain their neurotoxic effects on GM and functional connectivity through receptor-mediated mechanisms. As suggested in HSE, microglia may release cytokines such as these interleukins under the inflammatory phase. These cytokines can be responsible for glial cell loss and activation of the apoptotic cascade, which may contribute to the reduction in VBM grey matter volume and functional connectivity changes (Chen et al., 2019; Patrycy et al., 2022). These interleukins have also been associated with neurodegeneration and adverse effects on long-term potentiation in animal models, where increased levels of proinflammatory cytokines were present (Katzilieris-Petras et al., 2022; Bellinger et al., 1995).

We found a significant interaction between IL-6 and cerebral metrics (VBM grey matter volume and functional connectivity) on cognitive metrics in HSE, suggesting that increased IL-6 levels and changes in VBM grey matter volume and functional connectivity affect cognitive measures. In the context of HSE, increased IL-6 is observed in the CSF, indicating a robust inflammatory response to the viral infection within the central nervous system (Aurelius et al., 1994) has been suggested to be linked with cognitive dysfunction (Campbell et al., 1993); high levels of IL-6 are a sign that the body is reacting to the HSV that causes encephalitis. Similar mechanisms (inflammation) have been suggested to lead to reduced cognitive function in individuals with reduced VBM grey matter volume and changes in cerebral functional connectivity. Thus, the interaction between increased IL-6 levels and changes in cerebral metrics in HSE jointly influences cognitive function.

It is important to acknowledge that the cerebral alterations observed in critically ill HSE patients may not solely reflect the primary viral pathology. Limbic structures, including the hippocampus and posterior cingulate cortex, are exquisitely vulnerable to secondary insults such as global hypoxia and hypoperfusion, which are common complications in the ICU setting due to respiratory failure, shock, or the use of sedative medications. Thus, the contribution of such critical illness-related sequelae to the observed structural damage must be considered.

Our study includes a relatively large HSE cohort with fMRI and cognitive measures. Our findings were in line with previous studies, which strengthens the reliability of our findings. Nevertheless, our findings should be interpreted cautiously, and several limitations should be considered. Firstly, a retrospective and observational study design was used in the study. It is therefore imperative that these findings be prospectively validated. Furthermore, advanced MRI methods might be better suited to assessing brain integrity in this setting than standard MRI. In the future, we hope to investigate the clinical relevance of these innovative neuroimaging techniques in HSE studies. We also need to acknowledge cognitive function was assessed solely using the MoCA, a screening tool rather than a detailed neuropsychological battery. While feasible in acutely ill ICU patients, the MoCA has ceiling effects, limited sensitivity to HSE-typical deficits (e.g., amnesia, executive dysfunction, social cognition), and is vulnerable to confounding by delirium, fatigue, aphasia, and sedation during acute illness. These factors reduce its specificity for precise brain–behavior correlations. Future studies using comprehensive testing in the chronic phase are warranted.

In conclusion, we found that HSE was associated with significant changes in VBM grey matter volume and functional connectivity compared with controls. There were associations between cerebral changes seen in HSE and CSF parameters as well as cognitive measures. CSF interleukin 6 levels and cerebral changes were associated with cognitive measures in HSE. We emphasize the need for further research, including prospective studies, to validate these observational associations, explore potential causal mechanisms, and design more personalized strategies for these patients, based at least in part on cerebral MRI data.

## Data Availability

The raw data supporting the conclusions of this article will be made available by the authors, without undue reservation.

## Supplementary material

SUPPLEMENTARY FIGURE 1 Consolidated flow diagram of study participant selection and inclusion for the analysis of HSE and control groups.

SUPPLEMENTARY FIGURE 2 Box plot of specific difference data between HSE patients and normal control in VBM and FC; ^*^*p* < 0.05 and ^**^*p* < 0.01.

SUPPLEMENTARY FIGURE 3 The manifestations of HSE patients on conventional magnetic resonance sequences.

SUPPLEMENTARY FIGURE 4 Multivariable regression analysis of brain regions, cytokines, and cognitive function (MoCA).

SUPPLEMENTARY FIGURE 5 Multivariable regression analysis of CSF pressure, CSF protein, and brain regions.
