# Supplementary figures and images for: Voxel-based morphometry and functional connectivity changes are associated with cognitive function in herpes simplex virus encephalitis
Source: Front Neurosci. 2026 Jan 12;19:1714446. doi: 10.3389/fnins.2025.1714446 (PMC12833072; doi:10.3389/fnins.2025.1714446)

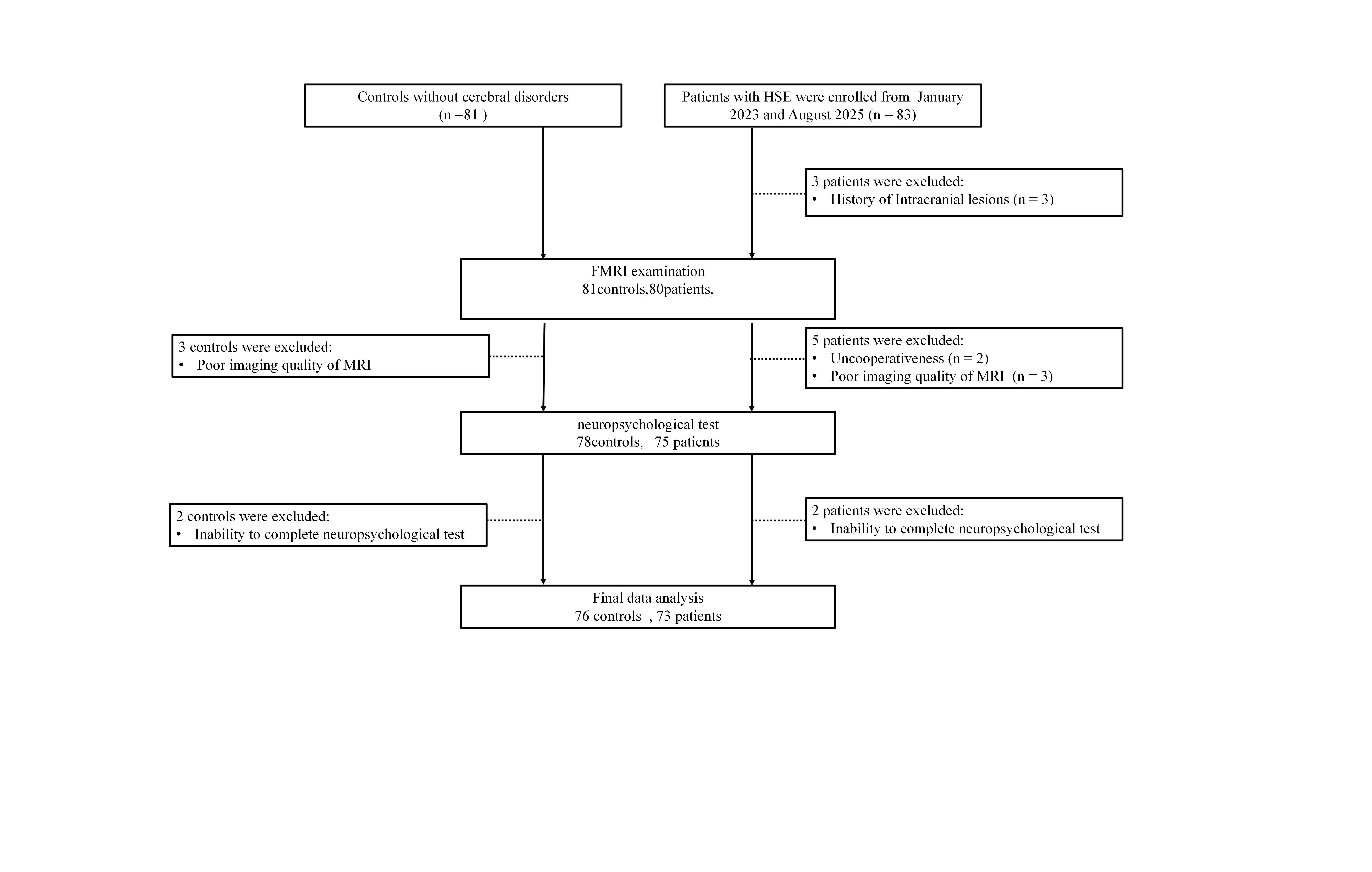

Supplement: SUPPLEMENTARY FIGURE 1 — Consolidated flow diagram of study participant selection and inclusion for the analysis of HSE and control groups. [file Image_1.jpeg]

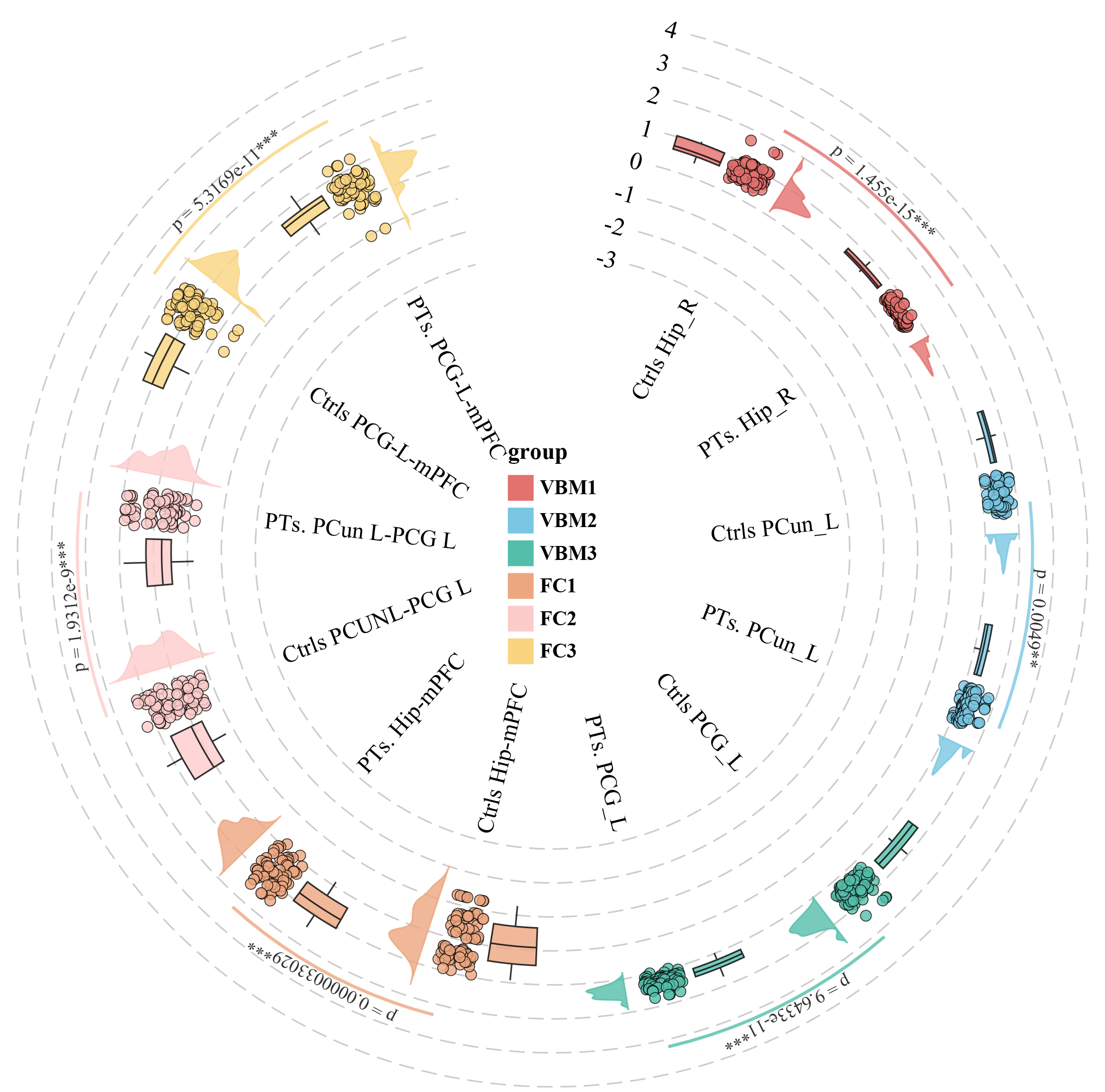

Supplement: SUPPLEMENTARY FIGURE 2 — Box plot of specific difference data between HSE patients and normal control in VBM and FC; *p < 0.05 and **p < 0.01. [file Image_2.jpeg]

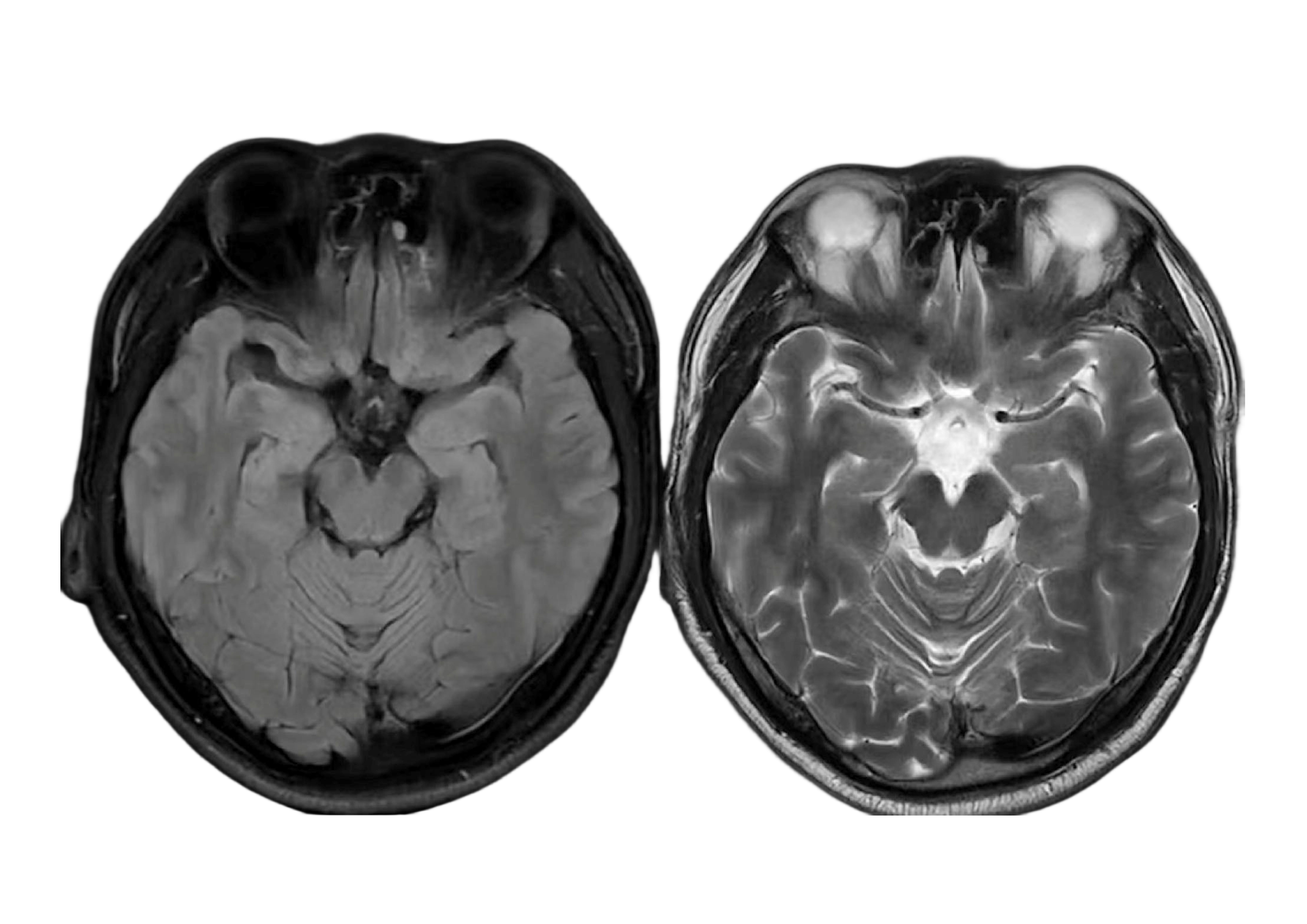

Supplement: SUPPLEMENTARY FIGURE 3 — The manifestations of HSE patients on conventional magnetic resonance sequences. [file Image_3.jpeg]

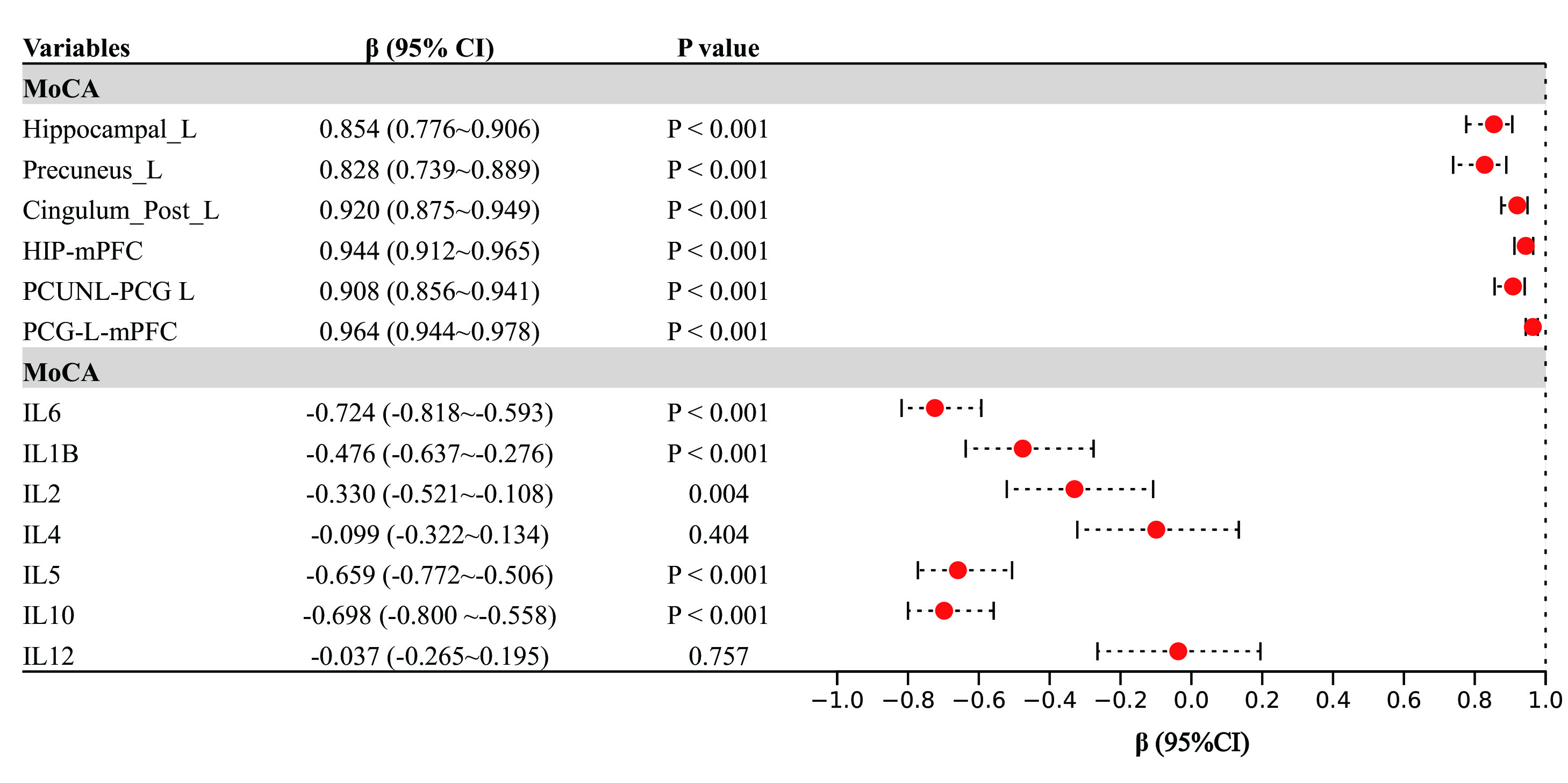

Supplement: SUPPLEMENTARY FIGURE 4 — Multivariable regression analysis of brain regions, cytokines, and cognitive function (MoCA). [file Image_4.jpeg]

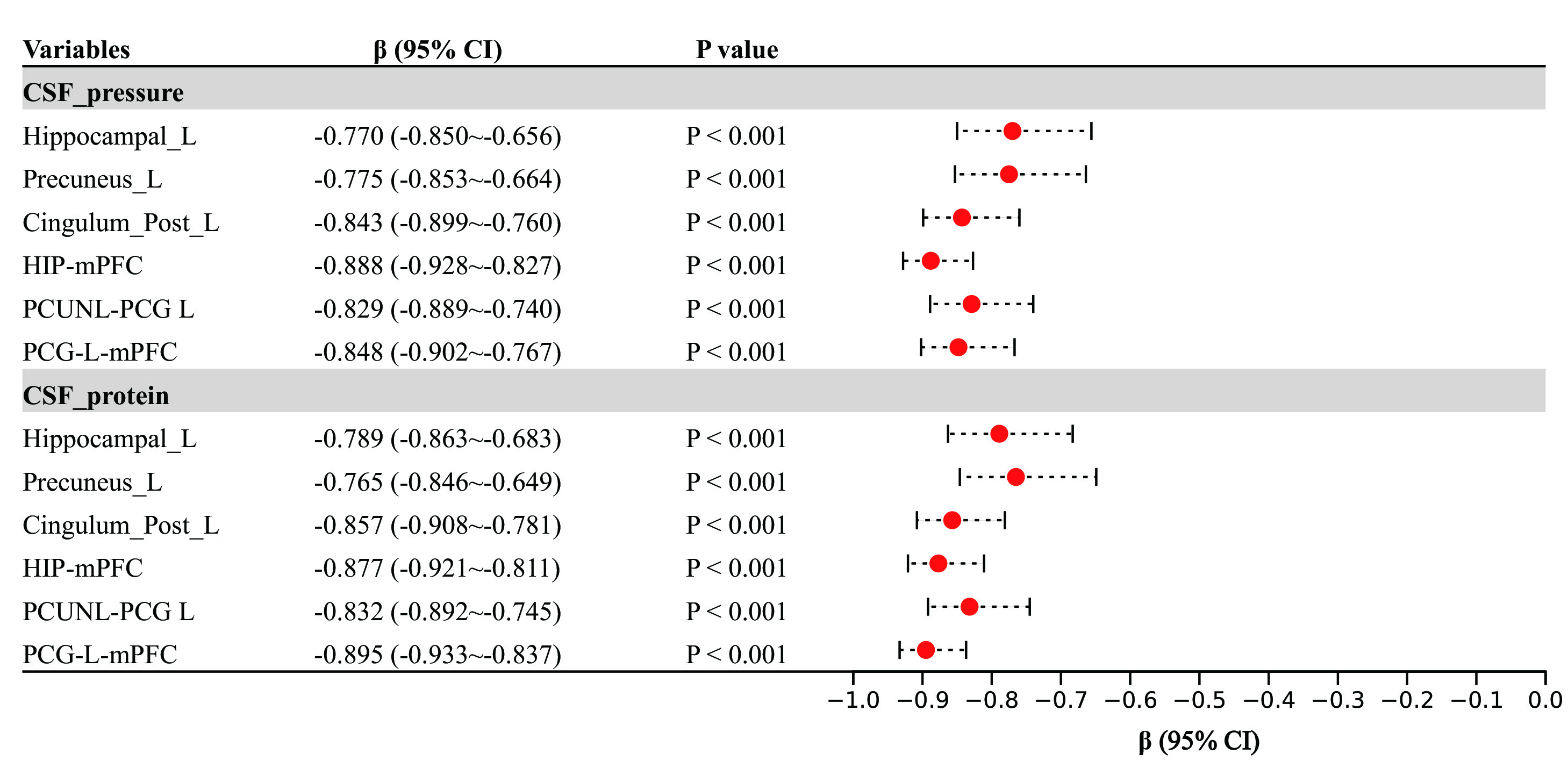

Supplement: SUPPLEMENTARY FIGURE 5 — Multivariable regression analysis of CSF pressure, CSF protein, and brain regions. [file Image_5.jpeg]
