# Supplementary material for: Voxel-based morphometry and functional connectivity changes are associated with cognitive function in herpes simplex virus encephalitis
Source: Front Neurosci. 2026 Jan 12;19:1714446. doi: 10.3389/fnins.2025.1714446 (PMC12833072; doi:10.3389/fnins.2025.1714446)
Supplement: Supplementary file 6 [file Table_1.docx]

| **Brain regions** | **Voxels** | **MNI coordinates** | | | ***P* value** |
| --- | --- | --- | --- | --- | --- |
|  |  | X | Y | Z |  |
| Hippocampal gyrus_R | 16 | 28 | -19 | -10 | <0.005 |
| Precuneus_L | 71 | -9 | -69 | 36 | <0.005 |
| Cingulate_Post_L | 49 | -6 | -45 | 33 | <0.005 |

## Brain regions with significantly decreased GMV in HSE compared to controls.
