# Supplementary material for: Voxel-based morphometry and functional connectivity changes are associated with cognitive function in herpes simplex virus encephalitis
Source: Front Neurosci. 2026 Jan 12;19:1714446. doi: 10.3389/fnins.2025.1714446 (PMC12833072; doi:10.3389/fnins.2025.1714446)
Supplement: Supplementary file 7 [file Table_2.docx]

# Original vs. FD-covaried FC statistics.

| **Group** | **N** | **Mean FD ± SD (mm)** | **p (t-test)** |
| --- | --- | --- | --- |
| Controls | 76 | 0.11 ± 0.03 | — |
| HSE | 73 | 0.13 ± 0.05 | **0.021** |
