# Supplementary material for: Voxel-based morphometry and functional connectivity changes are associated with cognitive function in herpes simplex virus encephalitis
Source: Front Neurosci. 2026 Jan 12;19:1714446. doi: 10.3389/fnins.2025.1714446 (PMC12833072; doi:10.3389/fnins.2025.1714446)
Supplement: Supplementary file 8 [file Table_3.docx]

# Table S3. Lesion Distribution on Routine Structural MRI in HSE Patients (N = 73)

| Brain Region Involved | No. of Patients (%) |
| --- | --- |
| Medial temporal lobe | 42(57.5%) |
| Insular cortex | 39 (53.4%) |
| Basal frontal region | 32 (43.8%) |
| Thalamus | 27 (36.9%) |
| Occipital lobe | 19 (39.7%) |
| Bilateral involvement | 29 (39.7%) |
| No visible lesion on T1/FLAIR | 3 (4.1%) |
